# Changing trends on the place of delivery: why do Nepali women give birth at home?

**DOI:** 10.1186/1742-4755-9-25

**Published:** 2012-10-10

**Authors:** Saraswoti Kumari Shrestha, Bilkis Banu, Khursida Khanom, Liaquat Ali, Narbada Thapa, Babill Stray-Pedersen, Bhimsen Devkota

**Affiliations:** 1 Department of Health Promotion and Health Education, Public Health, Bangladesh Institute of Health Sciences, Dhaka, Bangladesh; 2 Department of Community Medicine, Nepal Army Institute of Health Sciences, Kathmandu, Nepal; 3 Division of Women and Children, Oslo University Hospital Riks hospital and University of Oslo, Oslo, Norway; 4 Department of Health Educations, Faculty of Education, Tribhuvan University, Kathmandu, Nepal

**Keywords:** Home delivery, Institutional delivery, Changing trends, Nepal

## Abstract

**Background:**

Home delivery in unhygienic environment is common in Nepal. This study aimed to identify whether practice of delivery is changing over time and to explore the factors contributing to women’s decision for choice of place of delivery.

**Methods:**

A community based cross sectional study was conducted among 732 married women of reproductive age (MWRA) in Kavrepalanchok district of Nepal in 2011. Study wards were selected randomly and all MWRA residing in the selected wards were interviewed. Data were collected through pre-tested interviewer administered questionnaire. Chi-square and multivariate analysis was used to examine the association between socio-demographic factors and place of delivery.

**Results:**

The study shows that there was almost 50% increasement in institutional delivery over the past ten years. The percentage of last birth delivered in health institution has increased from 33.7% before 10 years to 63.8% in the past 5 years. However, the place of delivery varied according to residence. In urban area, most women 72.3% delivered in health institutions while only 35% women in rural and 17.5% in remote parts delivered in health institutions. The key socio-demographic factors influencing choice of place of delivery included multi parity, teen-age pregnancy, less or no antenatal visits. Having a distant health center, difficult geographical terrain, lack of transportation, financial constraints and dominance of the mothers- in-law were the other main reasons for choosing a home delivery. Psychological vulnerability and insecurity of rural women also led to home delivery, as women were shy and embarrassed in visiting the health center.

**Conclusion:**

The trend of delivery at health institution was remarkably increased but there were strong differentials in urban–rural residency and low social status of women. Shyness, dominance of mothers in law and ignorance was one of the main reasons contributing to home delivery.

## Background

A report published by UN agency had mentioned an estimated 358,000 maternal deaths occurred worldwide in 2008 [1], this figure showed 34% decline from the level of 1990 [2]. Despite this decline low income countries continue to account for 99% of maternal deaths primarily in Africa and South Asia [3]. Maternal mortality rate (MMR) shows a wide gap between rich and poor countries. Among developing regions South Asia has the second highest MMR at 280 maternal deaths per 100,000 live births in the global context [2]. The place of delivery is a crucial factor which affects the health and well-being of mother and newborn [4]. The percentage of birth attended by skilled health workers remains lower in South Asia i.e. 45% as compared to other Asian regions [5]. The percentage of institutional delivery was 20% in Nepal whereas 97% in Sri Lanka, and 39% in India [6].

In Nepal, MMR reported as 281 deaths per 100,000 live births [7]. Ministry of Health and Population has estimated that nearly 4500 women die every year from pregnancy related complications [8], mostly because of lack of skilled birth attendants and the absence of emergency services and equipments in rural health centers [9]. The vast majority (73%) of birth takes place at home in rural area of Nepal among them 55% of women are assisted by traditional birth attendants and relatives [10]. Some 40% deaths occur at home, 14% in transit to health facilities and 41% in health facilities [11]. A retrospective study done in Nepal has mentioned the major complications were retained placenta 84.1%, postpartum hemorrhage 17%, shock 10.2% and third degree perineal tear, the study revealed that home deliveries are associated with increased maternal morbidity related to the third stage complications [12]. Nepal had made an effort to achieve the Millennium Development Goal (MDG-5) targeted for reducing MMR by three quarter to 134 per 100,000 live births by 2015 [13]. The MDG report showed little improvement in decreasing MMR in Nepal [14]. The achievement however is not uniform across the rural and urban setting and there seemed to be important disparities by caste/ethnicity, socio-economic and eco-geographical regions [15]. Proper medical attention and hygienic condition during delivery can reduce the risk of complications of mother and baby.

The Government of Nepal lunched free delivery services at any public health facilities in 2009, with safe delivery incentive program already in place that aims to save maternal and new born lives by encouraging more women to deliver their babies in health facilities [16]. Despite this still women deliver their babies at home in unhygienic condition. In the above context this study aims to identify the changing trends of delivery practices in terms of place and person in different time intervals and to explore the reasons why mothers prefer to give birth at home.

## Methodology

### Study area

The study was conducted in Kavrepalanchok district of Nepal. The district was purposively selected as a hilly district of central Nepal. Estimated population of the district was 443,886 in 2008 [17]. This population covers 1.6% of the national population. The district has two private hospitals, one teaching hospital, four primary health care centers (PHCC), nine health posts (HP), eighty sub-health posts (SHP) and twenty five birthing centers. PHCC, HP, SHP and birthing centers are the different levels of community health centers. The reported MMR of the districts was 281 per 100,000 live births and 36.06% of delivery took place in health institutions [17].

A community based cross-sectional study was conducted during the period of Nov 2010-July 2011. Multi-stage random sampling was carried out for data collection in three steps. At the same time out of seventy five districts, majority (n=39) of the districts are the hilly districts [18]. The district has three municipalities and eighty nine village development committees (VDC) [19]. Among them Banepa municipality, Baluwa and Mechchhe VDCs were purposively selected as convenient sampling. Then two wards from each municipality and VDCs were selected as study area through simple random sampling using lottery method. Wards were the smallest unit of the study area. Banepa municipality is an urban area, which is 4 km away from the district head quarter with the majority of Newar ethnicity. Likewise Baluwa VDC, 18 km far, was selected as a rural area with majority of Danuwar ethnicity who are marginalized group in the Nepalese context. Similarly Mechchhe VDC was selected as a remote area of the district with the majority of Tamang ethnic group. It is 27 km far from the district head quarter [19].

All married women of reproductive age (MWRA) between 15–49 years residing in study wards were selected for the interview; they were 785 in total from which 53 were excluded because they never got pregnant. So all MWRA residing in study area who ever had birth were included and among them those who never had birth were excluded. In this study, delivery practices were the outcome of interest, two specific variables were examined, each defined as home and institutional delivery was dependent variable whereas independent variable was the individual characteristic of women that includes age, education, husband’s education, residence, ethnicity, parity and number of ANC visits. Data was collected by using semi-structured questionnaire after taking verbal consent. Formal approval was obtained from Nepal Health Research Council (NHRC) and a written permission obtained from the District Health Office (DHO), Kavrepalanchok and local authority of each VDC before conducting the study. However, it is important to consider the recall bias in mentioning the past history of delivery practice. Moreover time and resource constraints were the other limitations of the study. Percentage and frequency were used to describe the obtained socio-demographic information and χ2 and multivariate regression analysis was carried out to identify the association between different variables. Data were analyzed using statistical software SPSS version 11.5.

## Results

Table 1 represents the socio-demographic characteristics of the respondents. The mean age of respondent was 32.69±7.64. About 48.4% of the respondents represented from urban area, more than 38% from rural and 13% from remote area. Almost half (50.4%) of the women were illiterate and majority (83.5%) mentioned themselves as housewives. In this study, the highest proportions of the respondents belong to Newar ethnic group and majority was Hindu by religion as well as 47% of women’s husbands had higher secondary and above level of education. Currently 10.9% women are found smoker and 31.1% consume alcohol. Likewise majority (76.4%) women had 1 to 3 (2.78± 1.68) number of pregnancy. The mean age of first pregnancy was 20.01±3.07. Almost half (49.1%) of women had delivered their last baby within the last five years.

**Table 1:** Socio-demographic characteristics of the respondents (n=732)

**Socio-demographic variables**	**Category**	**Number**	**%**	**M ±SD**
Age	< 19	8	1.1	32.69±7.64
	20-29	272	37.2	
	>30	452	61.6	
Residence	Remote Area	97	13.3	
	Rural Area	281	38.4	
	Urban Area	354	48.4	
Education	Illiterate	369	50.4	
	Primary-Secondary	174	23.8	
	Higher secondary^**+**^	189	25.8	
Occupation	House wife	611	83.5	
	Farming	22	3.0	
	Business/Service and Other	99	13.5	
Marital Status	Married	692	94.5	
	Widowed	25	3.4	
	Polygamy (Co-wife)	15	2.0	
Religion	Hindu	566	77.3	
	Baudha	153	20.9	
	Christian	13	1.8	
Ethnicity	Tamang/Danuwar and Others	213	29.1	
	Newar	294	40.2	
	Bramhan/Chhetri	225	30.7	
Types of family	Nuclear	440	60.1	
	Joint	292	39.9	
Smoking Habit	Currently Yes	80	10.9	
	Never Smoked	652	89.1	
Alcohol drinking Habit	Currently Yes	228	31.1	
	Never Drunk	504	68.9	
Husband Education	Illiterate	127	17.3	
	Primary-Secondary	262	35.8	
	Higher secondary^**+**^	343	46.9	
Number of Pregnancy	1-3	559	76.43	2.78±1.68
	4-6	144	19.7	
	>7	29	4.0	
Age at First Pregnancy	<19	344	47.0	20.01±3.07
	20-29	381	52.0	
	>30	7	1.0	
Delivery Period of Last Children	> 10 year	226	31.1	
	5-10 year	144	19.7	
	<5 year	362	49.1	
Total		732	100	

**Figure 1:**
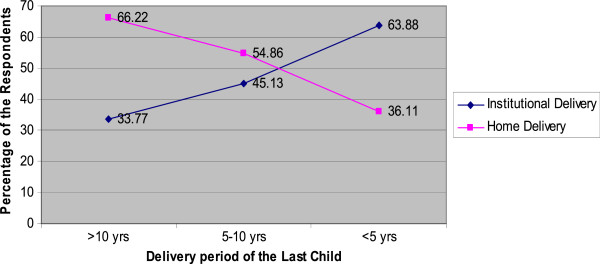
Changing Trends of the Delivery Place according to Last Delivery by 5 Years Interval (n=732).

Figure 1 shows the trends of delivery place in different time period. The majority (66.2%) of women had delivered their baby at home before 10 years. This trend has decreased to 54.8% during the period of 5 to 10 years. Similarly the trend was decreased up to 36.1% in last five yrs. On the other hand, the trend of institutional delivery has almost doubled as compared to before 10 years. Delivery in kitchen, bed room, animal shed, and other places rather than at a health center is considered as home delivery while institutional delivery is defined as a delivery that takes place at the government health institution as hospital, PHC, HP, SHP, birthing centers and private hospital or clinics.

**Figure 2:**
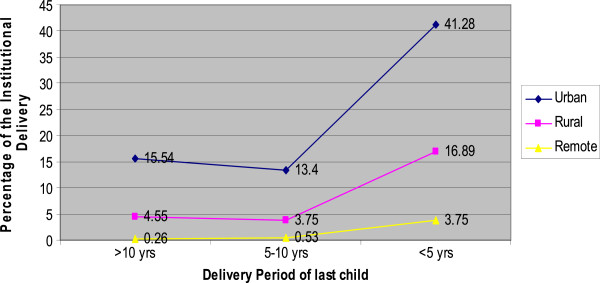
Residential differentials of Institutional Delivery in different Time Interval n= 373.

Figure 2 depicts the residential differentials of institutional delivery in different time period. The data shows almost 42% of the respondents from urban area had delivered their baby in health center whereas 17% women from rural and only 4% from remote area delivered in health center within the past five years preceding the study. There were remarkable differences between three different residences in different period of times.

Table 2 presents the changing trends of delivery practices in terms of place of delivery, delivery attendance and cord cutting instruments in different time intervals. The data shows that 28.0% of women had delivered their babies in kitchen before 10 years which was decreased to 19.7% in the recent 5 years. Likewise animal shed delivery also decreased by half (3.8%) in recent years. Similarly delivery that took place in bedroom also decreased from 31.5% to 12.5%. At the same time delivery conducted by skilled birth attendance (SBA) also increased from 45.6% to 64.4%, and sterile blade as a cord cutting instrument increased to 66%. But unsterile blade, sickle and knife were still common in practice. Stone, bamboo stick, wood, thread and broken glasses were considered as the other cord cutting instruments.

**Table 2:** Changing trends of delivery practices of the respondents (n=732)

**S. No.**	**Delivery practices of last birth**	**Period of last birth**							
1	**Place of Delivery**	**>10 year**		**5-10 year**		**<5 year**		**Total**	
		**n**	**%**	**n**	**%**	**n**	**%**	**n**	**%**
	Kitchen	64	28.07	43	29.83	71	19.72	178	24.31
	Animal Shed	15	6.57	5	3.73	14	3.88	34	4.64
	Bedroom and Others	72	31.57	31	21.52	45	12.5	148	20.21
	Health Institutions	77	33.17	65	45.13	230	63.88	372	50.81
2	**Delivery Attendance**								
	None	12	5.26	7	4.86	10	2.77	29	3.96
	Mother in-law	86	37.71	50	34.72	87	24.16	223	30.46
	SBAs	79	34.64	70	48.61	232	64.44	381	52.04
	TBAs and Other	51	22.36	17	11.08	31	8.61	99	13.52
3	**Cord Cutting Instruments**								
	Sterile Blade	95	41.66	74	51.38	237	65.83	406	55.46
	Unsterile Blade	98	42.98	51	35.41	94	26.11	243	33.19
	Sickle, Knife and Other	35	15.35	19	13.19	29	8.05	83	11.33
	**Total**	228	100	144	100	360	100	732	100

Table 3 reveals the association between socio-demographic variables and place of delivery respondents who had delivered their babies within the last five years by χ2 analyses. There was also the association between place of delivery and socio-demographic variables i.e. education, ethnicity, residence, family type, number of pregnancy, age at first pregnancy, number of ANC visit and husband’s education were statistically significant at p=<.05.

**Table 3:** Association with socio-demographic variables and delivery place of respondents who had delivered their baby in last five years using χ^2 ^analysis (n=362)

**Socio-demographic factors**	**Total (n)**	**%**	**Home (n)**	**%**	**Institute (n)**	**%**	**χ**^**2**^**Value**	**P-value**
**Age**								
< 19	8	2.2	1	0.27	7	1.93	4.16	.125
20-29	238	65.7	79	21.82	159	43.92		
30+	116	32.0	48	13.25	68	18.78		
**Education of Respondents**								
Illiterate	137	37.8	77	21.27	60	16.57	55.90	*
Primary- Secondary	100	27.6	36	9.94	64	17.67		
Higher Secondary+	125	34.5	15	4.14	110	30.38		
**Residence**								
Remote Area	56	15.5	40	11.04	16	4.41	79.99	*
Rural Area	126	34.8	63	17.40	63	17.40		
Urban Area	180	49.7	25	6.90	155	42.81		
**Ethnicity**								
Tamang, Danuwar & Other	117	32.3	73	20.16	44	12.15	67.06	*
Newar	137	37.8	18	4.97	119	32.87		
Bramhin/Chhetri	108	29.8	37	10.22	71	19.61		
**Education of Husband**								
Illiterate	43	11.9	27	7.45	16	4.41	37.71	*
Primary-Secondary	128	35.4	60	16.57	68	18.78		
Higher Secondary+	191	52.8	41	11.32	150	41.66		
**Family Type**								
Nuclear	192	53.0	78	21.54	114	31.49	.03	*
Joint	170	47.0	50	13.81	120	31.14		
**No. of Pregnancy**								
1-3	309	85.4	94	25.96	215	59.39	24.77	*
4-6	45	12.4	27	7.45	18	4.97		
7+	8	2.2	7	1.93	1	0.27		
**Age at First Pregnancy**								
< 19	147	40.6	73	20.16	74	20.44	22.15	*
20-29	211	58.3	54	14.91	157	43.37		
30+	4	1.1	1	0.27	3	0.82		
**Number of ANC Visits**								
None	41	11.3	33	9.11	8	2.20	65.33	*
1-3 visits	75	20.7	40	11.04	35	9.66		
>4 visits	246	68.0	55	15.19	191	52.76		

*n* Number of subjects*,* * level of significance at p=<.05.

Table 4 presents the multivariate regression analysis between socio-demographic variables and place of delivery. The respondents from remote area were 2.8 times and the respondents from rural area were 2.3 times less likely to have had institutional delivery than urban area (p=<0.5). Likewise women from Newar ethnicity were 2.5 times and from Tamang and Danuwar and other community were 1.2 times less likely to practice institutional delivery as compared to Brahmin/Chhetri women. Similarly illiterate women were 2.6 times and women with primary-secondary education were 2.5 times less likely to seek institutional delivery (p=<0.5). The respondents who never visited ANC had 5.5 times and who visited 1–3 visits were 2 times less likely to have had institutional delivery than women who had > 4 ANC visit (p=<0.5).

**Table 4:** Association between socio-demographic variables and delivery practices using multivariate regression analysis (n=362)

**Socio-demographic characteristics**	**Regression coefficient**	**OR for Exp (β)**	**95% CI Lower-Upper**	**P-Value**
**Residence**				
Remote Area	1.03	2.81	1.08-7.30	.034*
Rural Area	0.86	2.37	1.21-4.65	.012
Urban Area	1			
**Ethnicity**				
Tamang/Danuwar & Others	0.25	1.29	0.63-2.62	.485
Newar	−0.94	2.56	1.19-5.55	.016*
Bramhin/Chhetri	1			
**Education**				
Illiterate	.98	2.66	1.18-6.01	.018*
Primary-Secondary	.91	2.50	1.13-5.53	.024*
Secondary+		1		
**Husband Education**				
Illiterate	0.80	2.23	0.87-5.69	.095
Primary-Secondary	0.29	1.34	0.70-2.54	.376
Secondary+		1		
**Number of ANC Visits**				
No Visit	1.71	5.53	2.12-14.41	.000*
1-3Visits	0.74	2.09	1.09-3.97	.025*
>4 Visits		1		

The reference category is: Institutional Delivery*, n* Number of subjects*,* * level of significance at p=<.05.

Table 5 presents the reasons behind the home delivery by the women. More than 32% of respondents do not perceive institutional delivery as necessary. Almost 30% of respondents mentioned that because of unavailability of transportation they had delivered their baby at home. Similarly 22.6% of women said that health center was too far, almost 9% women had not been to institutional delivery because of shyness and fear of institutional delivery and about 13% women admitted that their mothers-in-law did not allow them for institutional delivery.

**Table 5:** Reasons given for home delivery by the respondents (n=128)

**S. No.**	**Factors**	**Frequency (n)**	**Percentage (%)**
1.	Financial Constraints	6	4.68
2.	Far distance of Health Center	29	22.65
3.	No Transportation	38	29.68
4.	Perceived not Necessary	40	31.25
5.	Mother in-law did not allow to visit Health Center	17	13.28
6.	Shyness and fear of Hospital Delivery	11	8.59
	Total	128	100.0

## Discussion

There was a considerable increase in institutional delivery over the different period of time. It was almost doubled as compared to women who had delivered their last child before 10 years and within the last 5 years. Similarly, a study conducted in Nepal showed the institutional delivery has increased from 8% in 1996 to 18% in 2006 [20]. Likewise other study conducted in Maharastra, India stated that percentage of institutional delivery had increased in different time period [21]. This might be due to the various programs along with safe motherhood and free services for institutional delivery. Safe Delivery Incentive Program (SDIP) and establishment of birthing centers in rural areas plays a vital role to increase institutional delivery.

Practice of delivery varied according to the place of residence. Women who reside in urban area had more institutional delivery than women from rural and remote area. The current finding was supported by DHS report, 2006 that almost 48% of the children in urban areas were born in a health facility, compared to rural area, (with 14%) [7]. A study done in Nepal revealed that skilled birth assistant during delivery plays a major role in the reduction of maternal mortality and morbidity [22]. In our study almost 50% of women were assisted by unskilled persons. Women still delivered their babies in unhygienic condition and still practicing harmful instruments for cutting cord especially in rural and remote area. This might be because of the rural disadvantage characterized with poor or no education, transportation, harsher geographic conditions and unavailability of the maternal services.

The traditional view on delivery practice was one of the major contributing factors for home delivery. In Nepal especially in rural and remote area women still believe in faith and fatalism, accepting whatever the consequences will come. Distance between home and health center, difficult geographical territory, lack of transportation, financial constraints, household dominance of mothers-in-law are the main reported reasons behind home delivery. A qualitative study conducted in Nepal also mentioned that women have little or no power in their marital home and are almost entirely at the mercy of their mother in law’s perception of their pregnancy and delivery care needs [23]. Similarly a study done in Malawi has found that cultural factors are important for health care seeking behavior in all communities especially the influence from decision makers in the choice of place of delivery [24]. Likewise, distance and transport was found to be one of the most important determinants in the decision of not seeking modern health care [25]. Another study showed that many pregnant women do not even attempt to reach a facility for delivery by walking many kilometers. It is difficult in labor and impossible if labor starts at night, and transport means are also often unavailable. Those trying to reach a far-off facility often fail, and women with serious complications may die in route [26]. In addition to the physical obstacles, the finding of our study showed other kinds of barriers such as fear and feeling of insecurity towards institutional delivery especially the rural and remote women. They feel shy and embarrassed to receive services from health center.

The study findings reveal that lower the education of women, lower the likelihood of institutional delivery practice. A study done in Nepal showed that poor maternal education and multi parity were important independent factors in determining choice of home delivery [27]. DHS 2006 also indicated that there is a strong association between institutional delivery and mother’s education [7]. By ethnicity, Tamang, Danuwar and other ethnic groups had less institutional delivery practice than Bramhin/Chhetri. Another study done in Nepal also showed that Tamang ethnic groups were less likely to have had post natal care than Bramhin/Chhetri [28]. Thus finding of our study showed there are poor institutional delivery practices among Tamang, Danuwar and other ethnic groups.

Regarding relationship between (Antenatal Care) ANC visit and institutional delivery practice, women who visited four or more ANC were more likely to have had institutional delivery as compared to women who never visited ANC. Another study done in Nepal also found that around 90% of women who made four or more ANC visits delivered in hospital as compared to 18.18% of women who never made an ANC visit [29]. ANC, therefore, is seen as a pathway to the institutional delivery. Moreover, education of women, residency, ethnicity and number of ANC visits were significantly associated with delivery practices.

## Conclusion

The changing trends of delivery practices of married women of reproductive age (MWRA) were remarkably increased in different time period. The increased trend is seen almost doubled within last 5 years. There was a noticeable difference in delivery practices between urban, rural and remote area. Women from remote area were practicing less institutional delivery as compare to urban women. There was almost half of delivery attended by unskilled person and harmful instruments were frequently used as cord cutting instruments. Kitchen was still the preferable place for delivery.

This study concludes that Socio-demography factors have significant influence in making a decision and choosing a place of delivery. Education of women and husband, residence and ethnicity are the important determinant of the delivery practices. Multi parity, teen-age pregnancy, less ANC or no ANC visits also significantly lead to home delivery. Majority of the respondents mentioned that lack of transportation, distance to health center, common perception ignoring to deliver in health institution, financial constraints, dominance of mother in-law and shyness or embarrassment appear as the main reasons behind home delivery.

## Competing interests

The authors declare that they have no competing interests.

## Authors’ contributions

SKS designed the study, developed protocol, involved in data collection, analysis and drafted the manuscript for publication. NT and BB were involved in study design, analysis and preparation of the manuscript. BD, KK, LA and BS-P offered scientific advice, inputs and critique during the study design, data analysis and in preparing the manuscript. All authors have read and approved the final manuscript.
